# Evaluation of routinely reported surgical site infections against microbiological culture results: a tool to identify patient groups where diagnosis and treatment may be improved

**DOI:** 10.1186/1471-2334-9-176

**Published:** 2009-11-10

**Authors:** Marco Krukerink, Job Kievit, Perla J Marang-van de Mheen

**Affiliations:** 1 Department of Medical Decision Making, Leiden University Medical Centre, Leiden, The Netherlands; 2 Department of Surgery, Leiden University Medical Centre, Leiden, The Netherlands

## Abstract

**Background:**

Surgeons may improve their decision making by assessing the extent to which their initial clinical diagnosis of a surgical site infection (SSI) was supported by culture results. Aim of the present study was to evaluate routinely reported SSI by surgeons against microbiological culture results, to identify patient groups with lower agreement where decision making may be improved.

**Methods:**

701 admissions with SSI were reported by surgeons in a university medical centre in the period 1997-2005, which were retrospectively checked for microbiological culture results. Reporting a SSI was conditional on treatment being given (e.g. antibiotics) and was classified by severity. To identify specific patient groups, patients were classified according to the surgery group of the first operation during admission (e.g. trauma).

**Results:**

Of all reported SSI, 523 (74.6%) had a positive culture result, 102 (14.6%) a negative culture result and 76 (10.8%) were classified as unknown culture result (due to no culture taken). Given a known culture result, reported SSI with positive culture results less often concerned trauma patients (16% versus 26%, X^2 ^= 4.99 p = 0.03) and less severe SSI (49% versus 85%, X^2 ^= 10.11 p < 0.01) suggesting that a more conservative approach may be warranted in these patients. The trauma surgeons themselves perceived to have become too liberal in administering antibiotics (and reporting SSI).

**Conclusion:**

Routine reporting of SSI was mostly supported by culture results. However, this support was less often found in trauma patients and less severe SSI, thereby giving surgeons feedback that diagnosis and treatment may be improved in these cases.

## Background

Between 2%-5% of all patients undergoing extraabdominal operations and up to 20% of all patients undergoing intraabdominal operations will develop a surgical site infection (SSI) [1]. Patients who develop SSI have longer hospitalizations, are twice as likely to die, 60% more likely to spend time in an ICU, and more than five times more likely to be readmitted to the hospital. Reduction of the incidence of SSI can substantially decrease morbidity and mortality and reduce the economic burden for patients and hospitals [2,3]. For these reasons, both medical professionals and policy makers in hospitals are interested in monitoring SSI.

Several definitions exist to define a SSI, such as the Nosocomial Infection National Surveillance Service (NINSS), the presence of pus, and the ASEPSIS scoring method [4,5]. But the most commonly used definition is that as defined by the Centers for Disease Control and prevention (CDC) National Nosocomial Infection Survey (NNIS) [6]. The CDC NNIS definition includes a SSI if one out of four criteria is fulfilled: (1) purulent drainage (2) a positive culture result from wound swab (3) local symptoms and opened by a surgeon, unless culture result is negative (4) when the diagnosis is made by a surgeon or physician. The CDC NNIS definition is advocated to be adopted as the universal, single, definition of SSI [7], as it is known that even small differences in definition may result in large differences in outcome, making comparisons between definitions unreliable [6].

Problems may occur in everyday clinical practice when attending physicians cannot afford to wait for culture results to materialize before starting subsequent treatment. This is addressed by the CDC NNIS definition, as merely the diagnosis of the attending physician is sufficient to include it. Key to helping surgeons to improve their decision making is to learn from SSI reporting data in the past, and the extent to which their diagnosis was supported by culture results. Since 1997 the department of surgery of the Leiden University Medical Centre (LUMC) is reporting SSI, as part of their reporting system of adverse outcomes for all surgical admissions. Preliminary results of these data have shown an increase in reported SSI from 2002 onwards [8] and the question is whether these reported SSI are supported by culture results.

Aim of the present study was to evaluate routinely reported SSI by surgeons against microbiological culture results, to identify patient groups with lower agreement where decision making may be improved.

## Methods

### Patients and definitions

All 701 surgical admissions in which a SSI was diagnosed in the LUMC in the period 1997-2005 were included. All adverse outcomes, including all SSI are prospectively recorded throughout admission on separate forms by surgeons and residents as part of routine medical care [9]. These forms are discussed on a weekly basis by a team of surgeons and residents for validity and possible preventability of adverse outcomes. Adverse outcomes are reported if they are so harmful to the patient's health that treatment is needed and/or the outcome results in permanent damage [10]. A previous study has shown that such a routine reporting system is an effective method to identify adverse outcomes [11]. As a result, a SSI will be reported if diagnosed and treated by a surgeon (in addition to prophylactic antibiotic treatment being given according to protocol), without knowledge of culture results at the time of reporting since these are not available at that point.

SSI - as all adverse outcomes - are classified in one of the following categories of severity: (1) temporary health disadvantage, recovery without (re) operation; (2) recovery after (re) operation; (3) (probably) permanent damage or function loss; and (4) death [10]. In the present study, less severe SSI were defined as SSI with severity category 1, while severe SSI included all other severity categories. Although SSI are commonly differentiated between incisional and organ/space infections, this is not yet incorporated into the adverse outcome reporting so that these data are unavailable. Furthermore, surgical wound class [12] is not registered routinely by surgeons in our hospital or elsewhere in the Netherlands. Since findings during surgery determine the wound class which are not (completely) reported in patients medical records, it is not possible to collect these data retrospectively in a reliable way. These data are therefore unavailable for the present study.

To assess whether results differed for specific groups of surgical patients, under the care of different wards and groups of surgeons, procedures performed in surgical patients were classified into surgery groups, according to the list of Operations 1999, which is developed and used by the Association of Surgeons in the Netherlands [13,14]. Patients were classified according to the surgery group of the first operation during admission, indicating the type of operation (and therefore type of patient). Four main groups were thus distinguished: (cardio)vascular surgical patients, trauma patients, patients undergoing surgery in digestive or gastrointestinal tract, and all other surgical patients. Patients with different surgery groups within the first operation were classified in the group all other surgical patients. All subsequent surgery during (re)admission is also included, to enable classification of severity of SSI (and other adverse outcomes).

### Culture results

All admissions with a reported SSI were retrospectively checked for culture results recorded electronically in the Hospital Information System. SSI with positive culture results from either wound or blood, within thirty days after the operation date were included. The thirty day window was chosen according to the NNIS-criteria [6]. Positive cultures from unrelated locations, e.g. lung or urine were ignored and did not contribute to the data presented. Cultures showing no growth were recorded as negative.

SSI in patients with no cultures taken within that time frame were recorded as unknown. For these patients we checked the medical records for clinical symptoms/criteria of a SSI and/or subsequent treatment, as well as any other information possibly explaining the lack of a culture.

### Survey

To identify the surgeon's views on the diagnosis and treatment of SSI, a survey was conducted among all surgical staff. The survey was carried out by telephone by a single person (MK) using a structured format and a duration of approximately 10 minutes. All 23 surgeons on permanent staff responded.

The survey consisted of open-ended questions on diagnosis, treatment and risk factors of SSI. We first identified the criteria for diagnosis and subsequent treatment of a SSI as used by each surgeon. Each surgeon could mention multiple criteria, which were retrospectively classified into groups. Secondly, we asked whether the surgeons perceived antibiotic treatment policy to be liberal or not. Finally, we asked whether risk factors existed in which case they would give antibiotic treatment sooner than when the above criteria were met, and what these risk factors were. Each surgeon could mention multiple risk factors, which were retrospectively classified into groups.

The Medical Ethical Committee of the Leiden University Medical Centre declared that formal ethical approval and written informed consent was not needed for this study under Dutch law.

### Statistical analysis

SSI with known and unknown culture results were first compared on other (patient) characteristics to assess whether no cultures were obtained in a particular group of patients, type of SSI or time period which may bias our results. Chi-square tests were used for categorical variables and t-tests for continuous variables. Characteristics included were: age at admission, sex, time period, ASA [15] class of first operation during admission as a measure of the patient's health status at admission, severity of SSI and type of surgical patients as defined by surgery group. ASA class was categorised with ASA class 3-5 taken as a measure of high-risk patients [16]. Time period was categorised into recent years (2003-2005) and earlier years (1997-2002) based on the increase in reported SSI [8]. Type of patients were grouped into trauma patients, (cardio)vascular patients, patients having surgery on digestive or gastrointestinal tract, and all other patients.

Within the group with known culture results, we then compared SSI with positive and negative culture results on each of the characteristics mentioned above. Chi-square tests were used for categorical variables and t-tests for continuous variables. Consequently, logistic regression analysis was used to look for independent predictors of a positive culture result, adjusted for differences in the other characteristics. Continuous variables (such as age) were first examined to check whether there was a linear relation between the potential confounder and the outcome. Variables showing a non-linear relation with the outcome were categorised. A p-value of less than 0.05 was considered statistically significant in all analyses.

## Results

Of all admissions with reported SSI, 523 (74.6%) had a positive culture result, 102 (14.6%) a negative culture result and 76 (10.8%) were classified as unknown culture result. Table 1 shows differences between SSI with known and unknown culture results. SSI with unknown culture results were less often diagnosed in males, less often high-risk patients and more often concerned the less severe SSI that do not require reoperation. In other words, given a culture being taken, chances are higher that the SSI results in more morbidity (e.g. reoperation).

**Table 1:** Characteristics of 701 patient admissions with reported surgical site infection, and differences between those with known and unknown culture results (Leiden University Medical Centre, 1997-2005)

	Known N = 625	Unknown N = 76	Test of difference
% male patients	54.2%	38.2%	X^2 ^= 7.03 **p < 0.01**
Average age (SD)	57.1 (18.4)	60.4 (15.6)	t = -1.67 p = 0.10
% ASA class > = 3	34.1%	15.8%	X^2 ^= 10.40 **p < 0.01**
Period			
% in 2003-2005	43.7%	51.3%	X^2 ^= 1.60 p = 0.21
Type of patients			
% (cardio)vascular	14.9%	17.1%	X^2 ^= 0.26 p = 0.61
% trauma	17.8%	26.3%	X^2 ^= 3.26 p = 0.07
% digestive or gastrointestinal tract	44.8%	36.8%	X^2 ^= 1.74 p = 0.19
% other	22.6%	19.7%	X^2 ^= 0.31 p = 0.58
% less severe SSI	70.6%	82.9%	X^2 ^= 5.10 **p = 0.02**

We further examined these SSI with unknown culture results to explore the reasons for not obtaining a culture. Of the 76 reported SSI with unknown culture results, 18 (23.7%) were drained without a culture being taken, 24 (31.6%) only had redness of the skin and/or pain without any other symptoms, 6 (7.9%) were presented with serous fluid, redness, swelling and/or pain, and 3 (3.9%) had cellulitis. Given that SSI were only reported if treated, the question is whether antibiotic treatment was necessary in these patients with only redness of the skin without other symptoms. For all these patients with SSI, no culture was taken. One (1.3%) had a culture taken during the weekend, which was not processed for unknown reasons. For 10 (13.2%) the patient records provided no further information. For 14 (18.4%) patients with unknown culture results, the written patient records could not be obtained. This was due to various reasons e.g. because they were in circulation at the moment of inquiry. The latter group of patients did not differ from the other patients with unknown culture results on any of the characteristics in table 1 (data not shown) so that it can be assumed that the reasons for not taking a culture were also similar in these patients. The SSI with unknown culture results were excluded from further analyses, thereby leaving 625 patient admissions.

### Culture results

Comparing SSI with positive and negative culture results, positive culture results more often concerned SSI reported in high-risk patients (table 2), which may represent the higher risk of infections in this group of patients. However, it is also shown that positive culture results less often concerned SSI reported in trauma patients and the less severe SSI (table 2). The finding that positive culture results less often concerned trauma patients was only found for the period 2003-2005 (18.8% versus 44.0%, X^2 ^= 14.41 p < 0.01) and not for the period before (14.3% versus 7.7%, X^2 ^= 1.69 p = 0.19). Furthermore, the finding that positive culture results less often concerned the less severe SSI was only found in trauma patients (49.4% versus 84.6%, X^2 ^= 10.11 p < 0.01) and not in other patients (72.6% versus 77.6%, X^2 ^= 0.84 p = 0.36). This suggests that the diagnosis of SSI in trauma patients may be improved, in particular when suspecting a minor SSI, i.e. by following a more conservative approach and not administering antibiotics directly. These results did not change when adjusted for differences in the other characteristics: the probability of a positive culture result was 45% lower in trauma patients (Odds Ratio 0.55 [0.33-0.94]), 44% lower in less severe SSI (Odds Ratio 0.56 [0.33-0.95]) and 77% higher in high-risk patients (Odds Ratio 1.77 [1.07-2.95]).

**Table 2:** Characteristics of patient admissions with reported surgical site infection: differences between those with positive and negative culture results (Leiden University Medical Centre, 1997-2005)

	Positive N = 523	Negative N = 102	Test of difference
% male patients	55.1%	50.0%	X^2 ^= 0.88 p = 0.35
Average age (SD)	57.0 (18.1)	57.7 (20.2)	t = 0.35 p = 0.73
% ASA class > = 3	36.1%	23.5%	X^2 ^= 6.04 **p = 0.01**
Period			
% in 2003-2005	42.6%	49.0%	X^2 ^= 1.41 p = 0.24
Type of patients			
% (cardio)vascular	15.1%	13.7%	X^2 ^= 0.13 p = 0.72
% trauma	16.3%	25.5%	X^2 ^= 4.99 **p = 0.03**
% digestive or gastrointestinal tract	45.9%	39.2%	X^2 ^= 1.54 p = 0.22
% other	22.8%	21.6%	X^2 ^= 0.07 p = 0.79
% less severe SSI	68.8%	79.4%	X^2 ^= 4.60 **p = 0.03**

### Survey

A survey among surgeons was conducted when they were unaware of the results mentioned above. The survey showed that most surgeons agreed on the clinical symptoms based on which they would report a SSI: redness/swelling was mentioned by 100%, pus by 65.2%, pain by 65.2% and fever by 60.9%. Of all surgeons, 73.9% reported that they did not always take a wound swab when a SSI was suspected, e.g. when only redness of the skin was observed (34.8%) and/or when there was no material to be collected (26.1%). Trauma surgeons on average mentioned more criteria than other surgeons (3.0 vs 2.4), in particular fever which was mentioned by 75.0% of the trauma surgeons vs. 57.9% of the other surgeons. This may indicate that trauma surgeons use a lower threshold or different criteria to diagnose a SSI, and may initiate antibiotic treatment sooner than other surgeons.

Antibiotic treatment was reported to be initiated sooner in the presence of osteosynthetic or prosthetic material (69.6%), immunosuppressed patients (60.9%), heart valve pathology (34.8%) and/or diabetes (21.7%). When a SSI was suspected without additional sickness or risk factors, treatment consisted of drainage only. Trauma surgeons on average mentioned the same number of risk factors for an earlier initiation of antibiotic treatment than other surgeons (2.3 vs 2.4). When asked specifically about antibiotic policy, three out of four trauma surgeons perceived their antibiotic policy to have become very liberal over time (against 1 out of 7 gastroenterologic/oncologic surgeons). It was not possible to test whether prescription behaviour had actually changed, since medication data were only available in the hospital information system from 2004 onwards. These survey results thus support the interpretation of the data above that a more conservative approach may be warranted in trauma patients.

## Discussion

The present study has shown that the majority (75%) of reported SSI had a positive culture result. These positive culture results more often concerned patients that were high-risk at admission, consistent with the general notion of chances of infection in patients with diminished health. However, positive culture results less often concerned trauma patients and less severe SSI, thereby suggesting that a more conservative approach with respect to treatment of suspected SSI may be warranted in these groups. In this way, routine reporting of SSI may help to identify such groups, and thus give valuable feedback to surgeons.

Our results seem to be similar to those from other studies. Our annual reported SSI incidence varies between 2.3% and 3.3% [8], within the range shown by other studies. In the UK 168 hospitals participated in the Nosocomial Infection National Surveillance Service (NINSS), showing that 4% of all admissions were complicated by a SSI, although the incidence varied considerably across hospitals [17]. The CDC NNIS estimates an incidence of 2.6% of SSI in the United States [16]. With respect to culture results, our percentage of 75% was lower than described by Giacometti et al. [18] where 91% of all 676 patients who presented signs and symptoms indicative of SSI showed pathological bacteria present. This gap of 16% can be partially explained by the SSI with unknown cultures in our study. These are likely to include some positive cultures had a culture been taken, as the presence of clinical symptoms or the presence of pus in these patients makes a SSI likely even if unconfirmed by culture results. However, assuming that all of these unknown culture results would have had positive culture results, the percentage would still only be 85%, suggesting that the initial diagnosis of suspected SSI can be improved. This explanation is supported by the fact that approximately one-third of the patients with unknown culture results only had redness of the skin and/or pain without any other symptoms.

Our survey showed that surgeons may initiate antibiotic treatment sooner if prosthetic material is present in patients. Unfortunately, we did not have complete information for each patient whether prosthetic material was present or not. If surgeons initiate antibiotic treatment sooner - i.e. with only few symptoms - because of prosthetic material being present, culture results may more often come back negative. This may explain why negative culture results more often concerned trauma patients, since prosthetic material is relatively common in these patients. However, we would then expect to find similar results in vascular patients, given the commonly present vascular prostheses. Since this was not the case, it seems unlikely for this to be the entire explanation.

Different groups of patients were distinguished to explore which type of patients more often had unknown or negative culture results, thereby guiding decision making of the different surgical wards. Infectious risk factors may be different in these groups, which may bias the comparison between these groups. Whereas a complicated fracture is a risk factor for infection in trauma patients, comorbidity of the patient and previous (groin) surgery are important risk factors in vascular patients. Since such data were not available for the present study, the comparison of culture results between surgery groups should be interpreted as a signal that decision making in trauma patients may be improved, rather than as an explanatory factor. This interpretation of the data is supported by our findings in the survey where trauma surgeons themselves also perceived their antibiotic policy to be very liberal.

The finding that positive culture results less often concerned trauma patients was only found for the period 2003-2005 and not for earlier periods, suggesting that the increase in reported SSI from 2002 onwards [8] is most likely due to more SSI with negative culture results being reported in trauma patients, in particular the less severe SSI. This interpretation is consistent with our survey findings where trauma surgeons perceived their antibiotic policy to have become very liberal over time. This perceived change in antibiotic policy may have been (partly) due to the arrival of a new head of the trauma surgeons, who supported this liberal antibiotic policy, thereby creating a gradual cultural change towards increased prescription of antibiotics.

In diagnosing a SSI the physician is left with his clinical judgment as wound swabs take days to produce results, in contrast to other infections (pneumonia, or urinary tract infection) were either X-ray or a urinary sediment can complement the diagnosis directly. This undoubtedly leaves more subjectivity in diagnosing a SSI, which can be sensitive to changes over time as a result of perceived risk perception, staff composition and/or cultural changes within a department. Quicker diagnostic methods would prove to be a considerable advantage, but are currently not yet available for hospital practice. Clearer guidelines on e.g. which and how many symptoms should be present, may help physicians to diagnose a SSI. The English NINSS has already included this: two of the following criteria (pain or tenderness, redness, heat and localized swelling) need to be present to diagnose a SSI [19]. Such a higher threshold may be particularly useful in some patients as identified in the present study. The results of the present study have increased the focus on the criteria for diagnosis and treatment of SSI in our surgical department, in particular in trauma patients and in less severe SSI, e.g. in meetings discussing adverse outcomes and the extent to which these should have been treated differently. Routine reporting of SSI over time has thus helped to improve diagnosis and treatment of SSI, by giving feedback to surgeons in which patients a more conservative approach may be warranted, and in this way has improved quality of care for future patients.

## Conclusion

Routine reporting of SSI was mostly supported by culture results. However, this support was less often found in trauma patients and less severe SSI, thereby giving surgeons feedback that diagnosis and treatment may be improved in these cases.

## Abbreviations

SSI: Surgical Site Infection; ICU: Intensive Care Unit; CDC NNIS: Centers for Disease Control and prevention National Nosocomial Infection Survey; LUMC: Leiden University Medical Centre; NINSS: Nosocomial Infection National Surveillance Service.

## Competing interests

The authors declare that they have no competing interests.

## Authors' contributions

MK collected the data on culture results, conducted the survey, performed the analysis and wrote the draft version of the manuscript. JK helped to design the study, with the collection of data, interpreting the data and writing the manuscript. PJM-vdM designed the study, supervised the collection of data, performed the analysis, interpreted the data, and wrote the final manuscript. All authors read and approved the final manuscript

## Pre-publication history

The pre-publication history for this paper can be accessed here:

http://www.biomedcentral.com/1471-2334/9/176/prepub
